# The Impact of Levetiracetam and Valproate on Platelet Functions—A Double-Blind, Placebo-Controlled Crossover Study

**DOI:** 10.3390/jcm12030933

**Published:** 2023-01-25

**Authors:** Itziar Olaizola, Martin F. Brodde, Beate E. Kehrel, Stefan Evers

**Affiliations:** 1 Faculty of Medicine, University of Münster, 48149 Münster, Germany; 2 Department of Anaesthesiology and Operative Intensive Care, University of Münster, 48149 Münster, Germany; 3 Department of Neurology, Lindenbrunn Hospital, 31863 Coppenbrügge, Germany

**Keywords:** levetiracetam, platelet, valproate, fibrinogen, P-selectin

## Abstract

It is known that valproate inhibits platelet functions; however, the exact mechanisms are not clearly identified. We studied 12 healthy adult volunteers (1 female, 11 male; age range 31.7 ± 7.8 years) before and after valproate 500 mg and compared the results to levetiracetam 1000 mg as a control substance and placebo. The study had a crossover and double-blind design. A blood sample was taken before and 90 min after medication intake, because the times to maximum serum concentration (T_max_) are 1.5 h for levetiracetam and 1 to 3 h for valproate. We analysed changes in platelet, erythrocyte, and leukocyte cell count and in platelet functions (CD62 expression (P selectin), thrombin binding, and fibrinogen binding). We found no significant differences in all cell counts before and after different study drugs. After valproate intake, but not after placebo or levetiracetam intake, the fibrinogen binding significantly decreased and the CD62 expression significantly increased resulting in decreased platelet aggregation. Our data suggest that the platelet dysfunctions reported for valproate result from decreased fibrinogen binding and from increased CD62 expression. This phenomenon might be one reason for the increased bleeding risk under valproate and cannot be observed for levetiracetam.

## 1. Introduction

Epilepsy patients are of increased risk for injuries; in addition, epilepsy surgery is expanding. These two causes of possible bleeding led to a particular interest on the impact of different antiepileptic drugs (AED) on platelets. Some of the older AED are known to have an influence on platelet functions and the complex coagulation system [1]. In most of them, the mechanisms are unknown. For the newer AED, their effect on platelets and the coagulation system is less well known.

Valproate (VPA) is still the most widely prescribed AED worldwide. It is a broad-spectrum AED, being effective against all seizure types, and has generally been regarded as a first-choice agent for most forms of idiopathic and symptomatic generalized epilepsies [2]. Beside others, one of the most commonly reported adverse effects of VPA is haemorrhagic diathesis [3]. Hematologic adverse effects occur in 33 to 55% of patients with VPA plasma levels between 215 and 1000 µg/mL and vary in onset and severity [4,5]. They are mostly transient. A linear correlation is observed between the serum levels and macrocytosis, thrombocytopenia, suppression of the bone marrow, or Pelger–Huet anomaly; a cumulative effect after prolonged intake is possible. VPA can cause a decreased platelet count and aggregation and an ATP release impairment. These side effects can appear after a few months of therapy and with plasma VPA levels within the normal range [6]. Even when the mean plasma concentration of the drug is low, VPA can reduce the activity of the arachidonate cascade in platelets. VPA effectively inhibits the cyclooxygenase pathway and the synthesis of the strong platelet aggregator thromboxane A2 [7]. In most situations, even when highly clinically significant, they can be reversed with dosage reduction; drug discontinuation is rarely required [6,8,9].

Levetiracetam is one of the most frequently used newer drugs against epilepsy. It is approved for nearly all types of epilepsy as monotherapy and as adjunctive therapy. It can be used for nearly all indications from the age of 16 years onwards. For adjunctive therapy it is even approved from the age of one month onwards. Adverse events usually appear within the first month after treatment initiation, are not dose dependent, are mostly mild to moderate, and generally resolve without medication discontinuation [10]. No significant changes in haematology, hemipterology, and chemistry profiles or weight occurred [11,12,13].

Levetiracetam is a structural analogue of piracetam. Piracetam has been shown to inhibit platelet aggregation. Piracetam also possesses antithrombotic activity in vivo [14]. It is possible that levetiracetam has a subtle effect on megakaryocytes, producing a previously unappreciated bleeding diathesis. However, this has not been shown clinically. Multiple case reports have shown levetiracetam to cause both isolated thrombocytopenia and pancytopenia. The phenomenon has been seen in both the adult and paediatric population. It is unclear what co-morbidities put patients at a higher risk of levetiracetam-related blood dyscrasias, but immunodeficiency was identified as a possible contributor [15]. In a case report, levetiracetam seemed to inhibit thromboxane-dependent platelet activation and aggregation [16]. In an epilepsy surgery unit, a higher than expected association between levetiracetam treatment and haemorrhagic complications of epilepsy surgery in series of patients has been described [17].

We aimed to compare the impact of VPA and of levetiracetam on different platelet functions in a placebo-controlled, double-blind crossover study.

## 2. Methods

### 2.1. Subjects and Procedure

We enrolled 12 healthy subjects (1 female) with a mean age of 32 ± 8 years and a mean body weight of 75 ± 8 kg. Baseline blood pressure was 119 ± 13 mmHg (systolic) and 69 ± 13 mmHg (diastolic). All subjects were free from psychiatric or physical illness, and in particular never had symptoms or a family history suggesting epilepsy or bleeding disorders. None of the subjects had taken any medication affecting platelet functions for at least two weeks before the study, including smoking. Written informed consent was obtained from all participants. The study was approved by the local ethics committee.

Blood samples were taken from the antecubital vein directly before and 90 min after intake of a single study drug always in the morning between 10 am and 12 pm. Patients received in a random order levetiracetam 1000 mg, VPA 500 mg, and placebo (glucose) in a single dose; the compounds were generic tablets in a non-extend release form. All three compounds were encapsuled by the pharmacy of the University Hospital of Münster using a glucose capsule with one standard size. The drug was given in a double-blind way (blinded for the subject and the laboratory) and in a crossover design. Every subject received all study drugs in intervals of 14 days (i.e., in total three blood samples before a study drug and three blood samples 90 min after a study drug). This resulted in a wash-out period of 14 days.

### 2.2. Laboratory Methods

The blood was anticoagulated with trisodium citrate (9 parts of blood, 1 part of trisodium citrate 0.108 mol/L). Platelet-rich plasma (PRP) was prepared by centrifugation at 250 g for 10 min at room temperature. The platelets were adjusted with phosphate buffered saline (PBS) to 25,000/µL.

### 2.3. Flow Cytometry Studies

Samples were analysed using a FACScan flow cytometer (Becton Dickinson, Heidelberg, Germany) with excitation by an argon laser at 488 nm. The FACScan was used in a standard configuration with a 530-nm bandpass filter. Platelets were gated with an automatic gating system as provided by the manufacturer, and data were obtained from fluorescence channels in a logarithmic mode. Specific binding of anti CD62P-FITC/fibrinogen-FITC was calculated.

### 2.4. Binding of Fibrinogen

Platelets were preincubated for 5 min at room temperature with 150 µL/mL fibrinogen-FITC (saturating concentration). An amount of 100 µL of the adjusted platelet suspension was activated with the thrombin receptor-activating peptide (TRAP, 0–100 µM) at room temperature. The reaction was stopped after 3 min by fixation with 100 µL of 1% formaldehyde (in PBS). After 30 min platelets were washed, resuspended in 500 of PBS, and analysed by flow cytometry [18].

### 2.5. P-Selectin Expression

Platelets (100 µL) were activated with TRAP (sequence SFLLRN; Nova Biochem via Merck Chemicals, Heidelberg, Germany) for 3 min and fixed with formaldehyde. After washing platelets and resuspending in PBS, they were incubated with anti-CD62P-FITC MoAb (clone CLB/thromb/6) (10 µL/mL) (Becton-Dickinson, Heidelberg, Germany). After one hour, platelets were again washed in PBS and analysed by flow cytometry.

### 2.6. Thrombin Binding

The building of thrombin on the platelet surface was quantified by ELISA (American Diagnostica, Pfungstadt, Germany). The amount of generated thrombin was visualized by thrombin-substrate and identified after 3, 5, 10, 15, 20, 30, and 60 min.

### 2.7. Statistical Analysis

Data are presented as arithmetic mean with standard deviation or in percentage. The power analysis revealed that 12 subjects were necessary to detect a difference of at least 30% between verum and placebo with a power of 80%. Comparison between the laboratory data before and after drug intake was performed by Wilcoxon test after Bonferroni correction. Comparison between the measures for the three different study drugs was performed by Kruskal–Wallis analysis with Mann–Whitney-U test as post hoc test. Significance level was set at *p* = 0.05.

## 3. Results

Table 1 shows the data of the cell count before and 90 min after intake of study drugs. All values were within the laboratory normal range. There were no statistically significant differences between the measures for the three study compounds and between the data before and after drug intake as analysed by the Wilcoxon and Mann–Whitney U test.

Table 2 shows the data of the thrombin binding, fibrinogen-FITC binding, and P-selectin (CD62P) expression before and 90 min after intake of study drug. There were statistically significant differences between the measures before and after VPA intake for fibrinogen-FITC binding and P-selectin expression. There were no changes in thrombin binding for either levetiracetam other VPA. Further, we observed no significant effects of levetiracetam or placebo on any of the platelet function tests.

In Table 3, the side effects reported by the subjects are described. After levetiracetam intake, tiredness, and dizziness for 30 min up to 4 h were reported by 7 of the 12 subjects. In one case, gastrointestinal pain after the intake of levetiracetam was reported. After VPA intake, dizziness in 2 of 12 subjects and tremor in one case were noted. There was no side effect with placebo. All side effects were regarded as mild and transient.

## 4. Discussion

The present study analysed the effects of levetiracetam and VPA on platelet count and functions after the intake of a single dose of 1000 mg and 500 mg, respectively. The drugs were chosen as comparative drugs because they are widely used. VPA can induce thrombocytopenia and platelet dysfunctions [19,20]. Levetiracetam is structurally similar to piracetam, which has demonstrated effects on platelet functions (antiplatelet activity mainly by inhibition of platelet aggregation, and increased erythrocyte deformability by effects on the cell membrane) [14].

Two cases of haematological abnormalities in patients receiving levetiracetam treatment were reported [17]. The first patient received oxcarbazepine in addition to levetiracetam, whilst the second received co-medication with lamotrigine and topiramate. Moreover, in another report only two out of five cases of haemorrhagic complications which the investigators identified as possibly linked to antiepileptic treatment, involved levetiracetam treatment [21]. Another case was a 19-year-old woman who was receiving levetiracetam and who developed haemorrhagic diathesis with ecchymosis [22]. Haematological testing revealed the presence of type I von-Willebrand-disease with 24% ristocetin cofactor activity (normal range 50 to 200%). Withdrawal of levetiracetam and its substitution by lamotrigine was followed by an increase in the ristocetin cofactor activity over a four-week period. However, we did not find any studies on the pharmaceutical impact of levetiracetam on platelets. This is in line with the vast majority of studies on this issue.

In our study, we observed a significant increase in P selectin (CD62P) expression and a significant decrease of fibrinogen binding by VPA; blood cell count and thrombin binding were not affected by VPA as has also been shown in other studies [23]. No significant differences at all were observed before and after levetiracetam and placebo intake.

An association between the daily VPA dose and a low platelet cell count has been described by several investigators [20,24,25,26], but not by all [27,28]. Thrombocytopenia occurred more frequently with high serum VPA levels [29], and some [30,31] reported evidence of dose-related suppression of haematopoiesis. The pathophysiology of the thrombocytopenia induced by VPA is unclear. In some studies [32,33,34], bone marrow examination has repeatedly demonstrated normal or an increased number of megakaryocytes, suggesting increased peripheral platelet destruction. On the other hand, damage to the platelet membrane by VPA could be caused by decreased production of platelet malonyl dialdehyde [35]. Since we could not observe a decrease of platelet cell count, we conclude that a single dose of VPA is not sufficient to have an impact on haematopoiesis in healthy subjects.

The present study demonstrates a platelet function alteration by VPA. After receiving a single dose of VPA 500 mg, we observed some abnormalities in platelet aggregation. The fibrinogen binding test showed a significant reduction; the expression of P-selectin was significantly increased. Both changes result in a decreased platelet aggregability and, thus, in a higher incidence of bleeding diathesis or haemorrhage. No significant abnormality was observed in the aggregation induced by thrombin binding suggesting that thrombin binding is not affected by a single dose of VPA.

An impairment of ATP release of platelets and of platelet aggregation correlated with both VPA dosage and plasma levels was observed [6]. The altered platelet function does not appear to be specific to a single agonist. Thus, VPA seems to have a broad effect on platelet function. Moreover, like aspirin, VPA can be regarded as an inhibitor of arachidonic acid aggregation. In children receiving VPA, the decreased platelet aggregation induced by ADP and collagen is related to the impaired secretory exocytosis of ATP release from dense bodies [6]. A group of 27 adult epilepsy patients receiving VPA monotherapy had significant decreases in platelet aggregation values compared to control subjects [20]. There were significant differences in collagen, arachidonic acid, ADP release, and aggregation that correlated with both VPA dose and plasma concentration. Another study revealed that the aggregation induced by ADP, epinephrine, and thrombin was inhibited by VPA, and ristocetin and collagen induction resulted in a normal aggregation response [36]. This finding suggests that the inhibition of platelet aggregation is not dose dependent and that the decreased aggregation response might be present without thrombocytopenia. Other investigations have not demonstrated a consistent relationship between VPA dose and qualitative platelet function [19,24,32]. The different findings may be explained in part by differences in study design and types of treatment.

The exact mechanisms of the platelet dysfunction induced by VPA have not been clearly elucidated. One hypothesis suggests that the mechanism may involve the inhibition of the platelet arachidonate pathway by VPA [7]. Fibrinogen depletion [37,38,39,40], low von-Willebrand-factor antigen [41], and increased thrombin time [42] have all been associated with VPA therapy. A risk of perioperative blood loss could, however, not be demonstrated for VPA [43].

Our study has some limitations. First, we examined healthy subjects and not patients with epilepsy. We chose this design because we wanted to be sure to evaluate the physiological effect of VPA and levetiracetam on platelets. We cannot exclude that the impact of these AED on platelets is different in patients with epilepsy who might have an increased aggregability of platelets by seizures (e.g., stress by generalized tonic-clonic seizures) or by long-term intake of AED. Second, we only studied the effect of a single dose but not of a treatment with several doses. An accumulation of the substances might occur which can result in a different impact on platelet functions. Since both AED have a short half-life time and we studied the effect on T_max_, we believe that our results are comparable even to long-term treatment. However, the accumulation of metabolites or other sequelae of the drug metabolism can be the cause of adverse events and are only observed in prolonged administration of the drugs. Third, we did measure plasma levels. Therefore, some variations in the study results between subjects could be just due to variations in the plasma level. However, the plasma level of both substances is very stable within some hours after the first intake, we believe that this limitation is only of minor importance. Fourth, we measured before and after study drug intake but not during the absorption. Therefore, we are not able to conclude on the pharmacokinetics after intake.

In conclusion, our study suggests that levetiracetam has no relevant effect on blood cell counts and on platelet functions as measured by thrombin binding, fibrinogen binding, and P-selectin expression. Furthermore, our study suggests that VPA is associated with decreased platelet aggregability. We could relate this known effect of VPA to decreased fibrinogen binding and increased P-selectin expression. In future studies, the clinical relevance of these findings has to be examined.

## Figures and Tables

**Table 1:** Difference between cell counts before and 90 min after intake of study drugs presented as arithmetic mean and standard deviation. No significant differences as analysed by Wilcoxon test.

	Levetiracetam Before	After	Valproate Before	After	Placebo Before	After
Leukocytes/mL	5433 ± 534	5740 ± 738	5480 ± 611	5590 ± 872	5525 ± 873	5710 ± 869
Platelets/nL	267 ± 47	264 ± 55	260 ± 39	269 ± 43	271 ± 46	266 ± 41
Erythrocytes × 106/µL	4.2 ± 0.5	4.2 ± 0.5	4.2 ± 0.5	4.1 ± 0.3	4.1 ± 0.4	4.1 ± 0.4

**Table 2:** Difference between thrombin binding, fibrinogen-FITC, and P-selectin (CD62P) expression before and 90 min after intake of study drugs presented as percentage (baseline as 100%), arithmetic mean, and standard deviation. Statistical comparisons between baseline and measures 90 min after intake were performed with Wilcoxon test.

	Levetiracetam	Valproate	Placebo
Thrombin binding 30’	15% ± 56%	2% ± 20%	19% ± 30%
Thrombin binding 60′	9% ± 23%	6% ± 13%	8% ± 15%
Fibrinogen 25 TRAP	−15% ± 31%	**−23%** ± **20%** *	−12% ± 20%
Fibrinogen 50 TRAP	−15% ± 31%	**−24%** ± **21%** *	−9% ± 21%
Fibrinogen 100TRAP	−18% ± 42%	**−19%** ± **24%** ***	−5% ± 22%
P selectin 25 TRAP	29% ± 55%	44% ± 55%	32% ± 58%
P selectin 50 TRAP	50% ± 72%	**54%** ± **47%** **	43% ± 62%
P selectin 100 TRAP	39% ± 57%	**57%** ± **51%** **	47% ± 41%

* *p* < 0.012 as compared to placebo and to levetiracetam. ** *p* < 0.017 as compared to placebo. *** *p* < 0.028 as compared to placebo.

**Table 3:** Number of patients reporting adverse effects after the intake of the study drugs.

	Levetiracetam	Valproate	Placebo
No adverse events	4	10	12
Tiredness/Dizziness	7	2	-
Tremor	-	1	-
Gastrointestinal pain	1	-	-

## Data Availability

Original data are available upon reasonable request.
